# Oral Hygiene Practices and Use of Preventive Dental Products Among Six-Year-Old Children at School Entry: A Cross-Sectional Study

**DOI:** 10.7759/cureus.104950

**Published:** 2026-03-09

**Authors:** Belkisa Hodzic, Elmedin Bajric, Selam Omerkic, Minela Becirovic, Emir Becirovic

**Affiliations:** 1 Department of Dentistry, Public Health Institution "Health Center" Živinice, Živinice, BIH; 2 Pediatric Dentistry, Faculty of Dentistry, University of Sarajevo, Sarajevo, BIH; 3 Department of Dentistry, Dental Care Clinic, Tuzla, BIH; 4 Department of Nephrology, Internal Medicine Clinic, University Clinical Center, Tuzla, BIH; 5 Intensive Care Unit, Internal Medicine Clinic, University Clinical Center, Tuzla, BIH

**Keywords:** children, cross-sectional study, fluoride toothpaste, oral hygiene, parental awareness, preventive dentistry

## Abstract

Background

Appropriate oral hygiene practices established in early childhood are essential for preventing dental caries. Parental awareness and supervision during the early school years play a key role in the development of effective daily oral hygiene habits. However, data on oral hygiene practices and the use of preventive dental products among children at school entry remain limited in many primary healthcare settings.

Materials and methods

This cross-sectional study included 307 six-year-old children who attended routine school-entry dental examinations at the Department of Dentistry, Public Health Institution "Health Center" Živinice, Bosnia and Herzegovina, between September 2022 and September 2024. Parents completed a structured questionnaire on the timing of the child’s first dental visit, initiation of toothbrushing, caregiver involvement in daily oral hygiene, and use of mechanical and chemical oral hygiene products. Categorical variables were summarized using descriptive statistics, and differences in questionnaire responses were analyzed using the Pearson Chi-square (χ²) test, with a significance level of p < 0.05.

Results

Delayed initiation of the first dental visit and toothbrushing were commonly reported. Only a minority of children attended their first dental visit following eruption of the first tooth, while most began brushing after eruption of several primary teeth. Parental supervision of oral hygiene was inconsistent, and independent brushing from the beginning was reported in a subset of children. Although regular toothbrush and toothpaste use was nearly universal, the use of adjunctive preventive products was limited, and more than half of parents were unaware of the fluoride content of their children's toothpaste.

Conclusion

Oral hygiene practices among six-year-old children appear suboptimal, with delayed initiation of preventive care and limited parental awareness of fluoride use. Educational interventions aimed at improving caregiver knowledge and supervision of children’s oral hygiene practices may support the development of more effective preventive behaviors at school entry.

## Introduction

Oral hygiene behaviors established in early childhood are central to the prevention of dental caries and other plaque-associated oral diseases. Dental caries remains the most prevalent chronic condition in children and is closely linked to delayed initiation of preventive care, inadequate tooth brushing routines, and inconsistent use of fluoride toothpaste [1,2]. At school entry, many children still lack the manual dexterity and persistence required for effective plaque removal, making caregiver supervision a key determinant of daily oral hygiene quality.

Professional recommendations generally support the first dental visit within the first year of life, ideally following eruption of the first primary tooth, primarily to enable anticipatory guidance for caregivers regarding oral hygiene techniques, fluoride use, dietary practices, and preventive strategies [3,4]. Nevertheless, available evidence indicates that first dental attendance often occurs later and is frequently symptom-driven, typically due to pain or established carious lesions [3,5]. Such delayed engagement limits opportunities for early risk reduction and habit formation.

In addition to a toothbrush and toothpaste, adjunctive preventive products, such as dental floss and interdental brushes, may improve interproximal plaque control. However, their use in young children depends almost entirely on caregiver knowledge, motivation, and practical feasibility. Data on caregiver-reported oral hygiene practices and preventive product use among children at school entry remain limited in several transitional healthcare settings.

Therefore, this cross-sectional study aimed to describe caregiver-reported oral hygiene practices and the use of preventive dental products among six-year-old children undergoing routine school-entry dental screening, including the timing of the first dental visit, initiation of tooth brushing, caregiver involvement in daily oral hygiene, and the use of mechanical and chemical oral hygiene products.

## Materials and methods

This cross-sectional study was conducted between September 2022 and September 2024 at the Department of Dentistry at the Public Health Institution "Health Center" Živinice, Bosnia and Herzegovina. The study included 307 children aged six years who attended routine school-entry dental examinations as part of the mandatory preventive dental program within primary healthcare services. The study sample represents a consecutive sample of all eligible children who attended scheduled examinations during the study period and whose parents provided informed consent and completed the study questionnaire. Children from both urban and rural areas of the municipality were included during regularly scheduled examinations throughout the study period.

Eligible participants were children aged six years who attended the scheduled dental examination during the study period. Inclusion required written informed consent from a parent or legal guardian and completion of the study questionnaire. Children whose parents declined participation or submitted incomplete questionnaires were excluded from the final analysis.

Dental examinations were performed in a clinical setting by licensed dental practitioners as part of routine preventive screening procedures. Each child underwent a non-invasive clinical visual dental assessment using standard dental instruments and appropriate illumination, in accordance with established pediatric preventive examination protocols. These examinations were conducted as part of routine preventive screening, and standardized clinical oral health indices such as the dmft index, plaque index, or gingival index were not systematically recorded for research purposes.

At the time of examination, parents or legal guardians completed a structured questionnaire addressing the timing of the child’s first dental visit, initiation of toothbrushing, caregiver involvement in daily oral hygiene practices, and use of oral hygiene products. The questionnaire consisted of closed-ended questions designed to capture caregiver-reported oral hygiene behaviors and preventive practices in early childhood. The questionnaire was completed during the same visit as the dental examination, with clarification provided by dental staff when necessary.

The study protocol was reviewed and approved by the Ethics Committee of the Public Health Institution "Health Center" Živinice (Approval No. 02-6006/22). All procedures were conducted in accordance with the ethical principles outlined in the Declaration of Helsinki.

Statistical analysis 

Collected data were entered into Microsoft Excel 2023 for Windows (Microsoft Corporation, Redmond, WA, USA) for data management and preparation of descriptive statistics. Statistical analyses were performed using IBM SPSS Statistics for Windows, version 23 (IBM Corp., Armonk, NY, USA). Categorical variables derived from questionnaire responses were summarized as absolute frequencies (n) and percentages (%).

## Results

A total of 307 six-year-old children were included in the study following parental consent and completion of the questionnaire. The study population consisted of 161 males (52.4%) and 146 females (47.6%).

**Figure 1:**
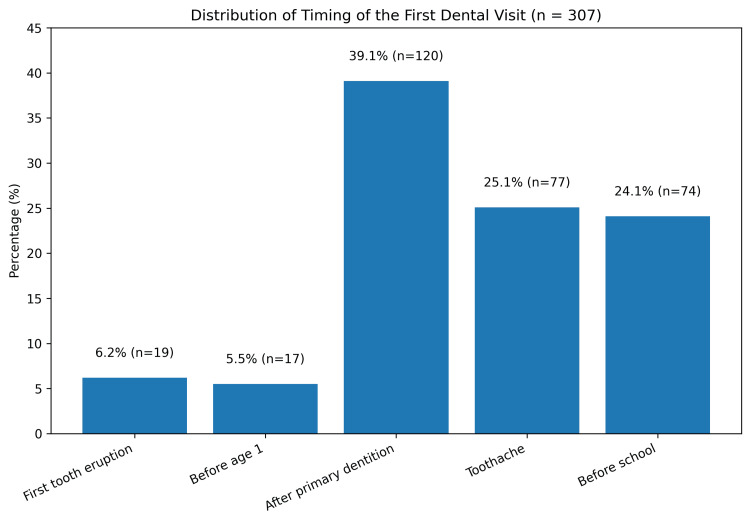
Distribution of timing of the first dental visit among six-year-old children Bar chart showing the distribution of parent-reported timing of the first dental visit among six-year-old children included in the study (n = 307). Values are presented as percentages.

Regarding the timing of the first dental visit, 19 children (6.2%) were taken to a dentist following the eruption of the first tooth, while 17 (5.5%) attended before the age of one year. A total of 120 children (39.1%) had their first dental visit after eruption of all primary teeth, 77 (25.1%) due to toothache, and 74 (24.1%) shortly before starting school. The distribution of responses regarding the timing of the first dental visit differed significantly across categories (χ² = 123.863, df = 4, p < 0.001) (Figure 1).

Regarding the initiation of tooth brushing, 79 children (25.7%) began brushing when the first tooth appeared, whereas 194 (63.2%) started after the eruption of several teeth. Brushing was initiated only after the eruption of all primary teeth in 29 children (9.4%), while five respondents (1.6%) reported other patterns of initiation. The distribution of responses regarding the initiation of tooth brushing differed significantly across categories (χ² = 275.971, df = 3, p < 0.001) (Table 1).

**Table 1:** Baseline characteristics and timing of oral hygiene initiation among six-year-old children Values are presented as numbers (n) and percentages (%). Percentages may not total 100% due to rounding.

Variable	n (%)
Gender	
Male	161 (52.4)
Female	146 (47.6)
Timing of first dental visit	
Following the eruption of the first tooth	19 (6.2)
Before the age of one year	17 (5.5)
After the eruption of all primary teeth	120 (39.1)
Due to a toothache	77 (25.1)
Shortly before starting school	74 (24.1)
Initiation of tooth brushing	
When the first tooth appeared	79 (25.7)
After the eruption of several teeth	194 (63.2)
After the eruption of all primary teeth	29 (9.4)
Other	5 (1.6)

Parental involvement in daily oral hygiene varied among participants. Parents reported being exclusively responsible for tooth brushing in 28 children (9.1%). In 129 cases (42.0%), children brushed with constant parental assistance, while 127 (41.4%) brushed with occasional help. Independent brushing from the beginning was reported in 23 children (7.5%). The distribution of responses across categories is presented descriptively.

All participants reported using a toothbrush as part of their daily oral hygiene. Additional oral hygiene aids were used less frequently, including wooden toothpicks (n = 48, 12.1%), interdental brushes (n = 19, 4.8%), dental floss (n = 20, 5.1%), and chewing sticks (n = 2, 0.5%). The distribution of responses regarding the use of oral hygiene aids (Table 2).

**Table 2:** Parental involvement in daily oral hygiene practices and use of preventive dental products among six-year-old children Values are presented as numbers (n) and percentages (%). Multiple responses were allowed for oral hygiene aid use; therefore, percentages may exceed 100%.

Variable	n (%)
Parental involvement in tooth brushing	
Parents exclusively responsible	28 (9.1)
Constant parental assistance	129 (42.0)
Occasional parental assistance	127 (41.4)
Independent brushing	23 (7.5)
Use of oral hygiene aids	
Toothbrush	307 (100.0)
Wooden toothpicks	48 (12.1)
Interdental brushes	19 (4.8)
Dental floss	20 (5.1)
Chewing sticks	2 (0.5)
Fluoride content in toothpaste	
Yes	85 (27.7)
No	64 (20.8)
Do not know	158 (51.5)

**Figure 2:**
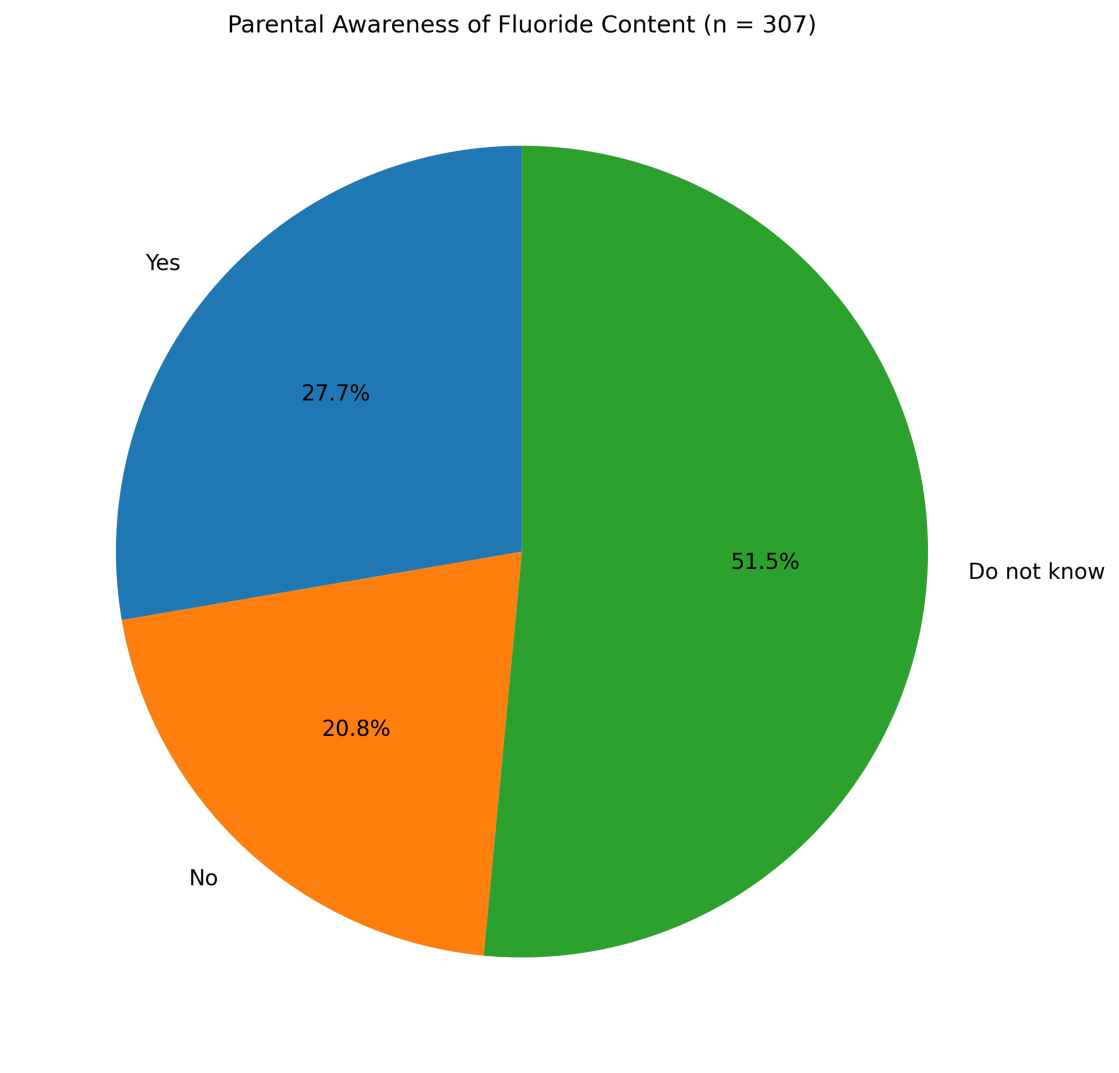
Parental awareness of fluoride content in children’s toothpaste Pie chart showing parental awareness regarding the fluoride content of toothpaste used by their children (n = 307). Values are presented as percentages.

Regular use of toothpaste was reported for 306 children (99.7%), while one child (0.3%) reported uncertainty regarding its use. Among respondents who used toothpaste, 85 (27.7%) reported that it contained fluoride, 64 (20.8%) reported using non-fluoridated toothpaste, and 158 (51.5%) were unaware of its fluoride content. The distribution of responses regarding awareness of fluoride content differed significantly across categories (χ² = 47.577, df = 2, p < 0.001) (Figure 2).

## Discussion

This cross-sectional study evaluated caregiver-reported oral hygiene practices and the use of preventive dental products among six-year-old children attending routine school-entry dental examinations in a primary healthcare setting. The findings indicate that initial contact with dental services in early childhood is frequently delayed beyond recommended guidelines and is often prompted by symptoms rather than preventive considerations.

Previous studies have reported that first dental visits in early childhood are commonly motivated by pain or established carious lesions rather than routine preventive assessment [3]. The present findings are consistent with these observations and suggest that early preventive dental attendance remains inconsistently implemented in this population. Delayed engagement with dental services may reduce opportunities for caregiver education on oral hygiene practices and dietary behaviors relevant to early childhood caries prevention.

The delayed initiation of tooth brushing observed in this cohort mirrors patterns reported in pediatric populations from Croatia and Jordan, where daily oral hygiene routines were often introduced only after the eruption of multiple primary teeth [6,7]. Insufficient mechanical plaque control during early childhood has been associated with increased susceptibility to dental caries and gingival inflammation in primary dentition.

Caregiver involvement in daily oral hygiene represents a key determinant of effective plaque removal in young children. Current preventive recommendations emphasize that tooth brushing in early childhood should be performed or closely supervised by caregivers, as children at this age generally lack the manual dexterity required for adequate plaque removal. Limited supervision during brushing may therefore contribute to suboptimal oral hygiene outcomes.

Although nearly all participants reported regular toothpaste use, caregiver awareness of fluoride content remained limited. Similar discrepancies between reported toothpaste use and knowledge of fluoride exposure have been described in studies evaluating parental oral health literacy [8]. Given the established role of fluoride in enhancing enamel remineralization and inhibiting demineralization processes, insufficient awareness of fluoride-containing products may diminish the preventive effect of daily oral hygiene practices.

The low utilization of interdental hygiene aids observed in this study may reflect limited caregiver knowledge or inadequate preventive counseling during early dental visits. Previous reports have shown that interdental cleaning improves plaque control and gingival health; however, its implementation in early childhood depends largely on caregiver instruction and motivation [5,9].

These findings underscore the importance of structured early caregiver counseling regarding supervised tooth brushing and the appropriate use of fluoride-containing toothpaste. In addition to individual-level education, integration of preventive oral health guidance into routine pediatric and school-entry healthcare services may represent an effective strategy for improving early oral hygiene behaviors. Strengthening caregiver engagement in preventive oral health practices during early childhood may contribute to improved long-term oral health outcomes in pediatric populations.

Limitations

The findings of this study should be interpreted in light of several limitations. Data were obtained using parent-reported questionnaires and may therefore be subject to recall bias or social desirability bias. The study was conducted at a single primary healthcare institution, which may limit the generalizability of the findings to other populations or healthcare settings.

The cross-sectional design precludes assessment of causal relationships between reported oral hygiene practices and clinical oral health outcomes. Additionally, the study did not include standardized clinical oral health indices such as the dmft index, plaque index, or gingival index. As a result, the study cannot directly evaluate the relationship between reported oral hygiene behaviors and objective oral health status. Furthermore, the statistical analysis was primarily descriptive, reflecting the observational and questionnaire-based nature of the study. Future studies incorporating both behavioral assessments and standardized clinical dental indices would provide a more comprehensive understanding of the association between oral hygiene practices and oral health outcomes in children.

## Conclusions

The findings of this study suggest that the first dental visit and the initiation of daily oral hygiene practices in early childhood are frequently delayed, while caregiver awareness regarding the preventive role of fluoride-containing toothpaste remains limited. Although regular toothbrush and toothpaste use was widely reported, the use of adjunctive preventive oral hygiene aids was uncommon.

Early preventive dental attendance and consistent caregiver-supervised oral hygiene practices may represent key targets for improving oral health behaviors at school entry. Structured educational interventions aimed at strengthening caregivers' knowledge of fluoride use and age-appropriate oral hygiene techniques could help establish more effective preventive practices in early childhood.
